# Pregnancy in Multiple Sclerosis: A Questionnaire Study

**DOI:** 10.1371/journal.pone.0099106

**Published:** 2014-06-05

**Authors:** Nadja Borisow, Friedemann Paul, Stephanie Ohlraun, Daniel Pach, Felix Fischer, Jan Dörr

**Affiliations:** 1 NeuroCure Clinical Research Center, Charité – Universitätsmedizin Berlin, Berlin, Germany; 2 Clinical and Experimental Multiple Sclerosis Research Center, Department of Neurology, Charité – Universitätsmedizin Berlin, Berlin, Germany; 3 Institute for Social Medicine, Epidemiology and Health Economics Charité, Charité – Universitätsmedizin Berlin, Berlin, Germany; 4 Department of Psychosomatic Medicine, Clinic for Internal Medicine, Charité – Universitätsmedizin Berlin, Berlin, Germany; Strasbourg university hospital, France

## Abstract

**Background:**

Multiple sclerosis (MS) preferentially affects females at childbearing age. For this reason patients and treating physicians were frequently confronted with questions concerning family planning, pregnancy and birth.

**Objective:**

The aim of this study was to evaluate the expertise about pregnancy related topics in multiple sclerosis of neurologists in private practice.

**Methods:**

We developed a survey with 16 multiple choice questions about pregnancy related topics and sent it to neurologists in private practice in Berlin, Germany.

**Results:**

56 completed questionnaires were sent back. 54% of all questions were answered correctly, 21% of the questions were answered with “I don’t know”. Correct answers were more often given by physicians who treat more than 400 MS patients per year (p = 0.001). Further positive associations were found for assumed relevance of the topic (p = 0.002) and the degree of counseling (p<0.001).

**Conclusion:**

To provide a comprehensive counseling, MS patients with desire for children should be counseled by physicians with a lot of experience in MS treatment.

## Introduction

Multiple sclerosis (MS) is a chronic autoimmune disease of the central nervous system which particularly affects young women. The male to female ratio ranges between 1∶2–3 [1], [2] and, while incidence seems to increase in general [3], [4], it seems to increase even stronger in women [5]. First symptoms typically arise between the third and fifth decade of life which overlaps with the period of reproduction and family planning. Assuming a prevalence in Europe of 70–80/100.000 during the third and fourth decade [1], there are estimated to be 250.000 female MS patients of fertile age in Europe. Hence, there is a great need of patients for counseling about inheritance risk, sexual dysfunctions, influence of MS on a pregnancy, birth and lactation, but also vice versa about the effect of pregnancy on the MS course.

In the past, especially during the first half of 20th century, female patients were often discouraged from becoming mother as pregnancy was considered to be a risk in MS [6]. Although some studies may suggest that MS increases the risk of adverse pregnancy outcomes [7], [8], the majority of studies could show that risk of complications during pregnancy and birth outcome is the same as in healthy women [9], [10]. Many patients in our outpatient department and in MS internet forums report that they received the advice to deliver by caesarean section although current studies show that it is not necessarily preferable to vaginal delivery in MS patients [11], [12]. Patients report to be frequently advised against peridural anesthesia, although it is well documented that peridural anaesthesia does not increase post partum relapse rate and can be used in MS patients too [13]. The problem of sexual dysfunction seems to be often underestimated as either patients do not report these symptoms or physicians do not routinely ask for them [14]. Briefly summarized, increasing data regarding MS and pregnancy has become available in the literature during the last years [15]. However, it is not known to what extent these findings are transferred into today’s clinical routine.

The uncertainties among patients and attending physicians also apply to handling of MS pharmacotherapy. Within the last years pregnancy registries could show that exposure to interferon beta (IFN-β) and glatiramer acetate (GA) during conception and even during pregnancy seems to be relatively safe [16], [17]. However, it is commonly agreed to discontinue IFN- β or GA treatment when pregnancy is detected. Limited data concerning pregnancy is available for the monoclonal antibody natalizumab and the sphingosine receptor modulator fingolimod [18], [19]. Mitoxantrone is known for teratogenic effects, and its withdrawal is recommended in female as well as in male patients at least six months before pregnancy. Until now, there are no reliable data whether it is favorable to start immunomodulatory therapy (IMT) directly after delivery instead of nursing or to postpone IMT in favor of breastfeeding [20], [21].

Taken together, MS patients that plan to conceive or are already pregnant have currently unmet needs in terms of guidance. As obstetricians are usually not experienced in MS counseling and, vice versa, neurologists have only little knowledge regarding obstetrics, pregnant MS patients may have difficulties to get competent advice.

This study surveys neurologists as main source of information for pregnant MS patients regarding delivery, course of the disease and medication during pregnancy, child birth and lactation.

## Methods

Prior to conducting our study we approached the institutional review board of the Charité – Universitätsmedizin Berlin and received approval for waiver of ethics committee vote.

We collected demographic and general data of the participants (e.g. age, sex, year of completing residency and number of treated MS patients). In Germany it is possible to pass board certification only in neurology or in both neurology and psychiatry; therefore we also asked for the individual specialization.

After a brought review of literature, we created a questionnaire containing 16 multiple choice questions about pregnancy related topics in MS. The questions were chosen among the issues that were brought up most often by patients in our outpatient department and that are topic of discussion in current literature [15]. These questions were designed as multiple choice items with single correct answer and “I don’t know” option; the language of the questionnaire was German. The questionnaire is available as supplement.

To evaluate the comprehensibility and time needed to complete the questionnaire, questions and possible answers of the survey were at first discussed and reviewed by neurologists and obstetricians not participating in the study. Approximately 15 minutes were needed to fill in the questionnaire.

For further analysis, the 16 questions were assigned to one of three categories.

1. family planning before conception
2. pregnancy and delivery
3. period after delivery.

Category 1 contains questions 1–5, 15 and 16 which deal with inheritance risk, frequency of sexual dysfunction, assisted reproduction technique, handling of immunomodulatory therapy during time of contemplating pregnancy, and influence of pregnancy on the long term course of MS.

Questions 6–11 address relapse rates during pregnancy, pregnancy associated complications, treatment of relapses during pregnancy, assisted delivery, peridural anaesthesia, and birth outcome in MS patients; they are summed up in category 2.

Category 3 encloses the questions 12–14 which deal with relapse rates after delivery, breast feeding, and immunomodulatory therapy during lactation.

The questionnaire was mailed to all neurologists in outpatient care in Berlin, registered at the Association of Statutory Health Insurance Physicians (Kassenärztliche Vereinigung). Physicians that treat mainly psychiatric patients (n = 101) were excluded from the study. One month later a reminder was sent. Participants received an expense allowance to the amount of 30 Euro. Anonymity was assured by using two envelopes: informed consent, accounting documents and the first envelope containing the completed questionnaire were placed inside a second envelope. In our institution the first envelope was stored separately from the other documents; therefore a personal allocation to specific persons was not possible.

Statistical analysis was performed by using SPSS Statistics version 19. To compare percentages, Kruskal-Wallis-test and Mann-Whitney-U-test were used for independent samples. Wilcoxon signed rank-test was used to compare related samples. Our study was an exploratory analysis, no power calculation had previously been performed, and not more than three groups were compared. Therefore no correction for multiple comparisons was performed.

## Results

### Demographic Characteristics

Out of 248 neurologists, 56 sent back the completed questionnaire (response rate 23%). Mean age of respondents (±SD) was 52 (±8.8) years. They passed board certification as a specialist in neurology on average 17 (±8.8) years ago and were in private practice for 11 (±9.2) years on average. 21 (37%) treat less than 10 MS patients per quarter, 24 (43%) attend 10–100 patients and 11 (20%) treat more than 100 MS patients per quarter. A summary of demographic data is presented in Table 1.

**Table 1:** Demographic characteristics.

Gender	male	33 (59%)
	female	23 (41%)
Age	<50 years	24 (43%)
	≥50 years	30 (54%)
	not specified	2 (3%)
Board certification	neurology	24 (43%)
	neurology & psychiatry	32 (57%)
Period of board certification	<15 years	24 (43%)
	≥15 years	31 (55%)
	not specified	1 (2%)
Private practice	<10 years	29 (52%)
	≥10 years	26 (46%)
	not specified	1 (2%)
Treated MS patients per quarter	<10	21 (37%)
	10–100	24 (43%)
	>100	11 (20%)
Counseling of female patients concerning pregnancy related topics	extensively	23 (41%)
	to a certain degree	28 (50%)
	not at all	5 (9%)
Counseling of male patients concerning pregnancy related topics	extensively	14 (25%)
	to a certain degree	35 (62%)
	not at all	7 (13%)
Relevance of pregnancy aspects in MS	very relevant	18 (32%)
	moderately relevant	25 (45%)
	little relevant	9 (16%)
	not relevant	3 (5%)
	not specified	1 (2%)

### Survey Results

About half (54%) of all questions were answered correctly. Among those who indicated to counsel MS patients about pregnancy related topics extensively or to a certain degree, the rate of correct answers was 59%.

The question which was most frequently answered correctly refers to MS relapse rate during pregnancy. 47 (84%) of the participants knew that relapse rate decreases during the course of pregnancy [13]. 42 (75%) were aware that the rate of pregnancy related complications in MS patients is the same as in healthy women. With 28 (50%) wrong answers the question about frequency of sexual dysfunctions in MS patients was the question most often answered incorrectly. Topics in which most of the participants were aware of their lack of knowledge refer to assisted delivery and artificial fertilization. 52% and 47%, respectively, answered these questions with “I don’t know”. Table 2 summarizes the survey results.

**Table 2:** Survey results.

	correct	incorrect	I don’t know	missing
Inheritance risk	31 (56%)	13 (23%)	9 (16%)	3 (5%)
Frequency of sexual dysfunctions	14 (25%)	28 (50%)	13 (23%)	1 (2%)
IMT before conception (female patients)	28 (50%)	19 (34%)	6 (11%)	3 (5%)
IMT before conception (male patients)	36 (64%)	8 (14%)	11 (20%)	1 (2%)
Escalation therapy before conception	14 (25%)	6 (11%)	11 (20%)	8 (14%)
Relapse rate during pregnancy	47 (84%)	3 (5%)	5 (9%)	1 (2%)
Complications during pregnancy	42 (75%)	7 (13%)	5 (9%)	2 (3%)
Relapse treatment during pregnancy	26 (46%)	18 (32%)	11 (20%)	1 (2%)
Assisted delivery	15 (27%)	11 (19%)	29 (52%)	1 (2%)
Peridural anaesthesie	41 (73%)	4 (7%)	10 (18%)	1 (2%)
Pregnancy outcome	35 (63%)	9 (16%)	10 (18%)	2 (3%)
Post partum relapse rate	37 (66%)	8 (14%)	8 (14%)	3 (5%)
Breast feeding	17 (31%)	27 (48%)	9 (16%)	3 (5%)
IMT post partum	33 (59%)	8 (14%)	7 (13%)	8 (14%)
Influence of pregnancies on longterm MS course	35 (63%)	3 (5%)	14 (25%)	4 (7%)
Assisted reproductive technique	12 (21%)	14 (25%)	26 (47%)	4 (7%)

IMT: immunomodulatory therapy.

After assigning the 16 questions to the three categories mentioned above, we found the most correct answers (68%) within category 2, which refers to the period of pregnancy and questions about delivery. The questions about topics before the beginning of pregnancy (category 1) were answered at least correctly (55%) and mostly with “I don’t know” (18%).

Table 3 summarizes the results of the statistical analysis. We compared the percentages of correct and “I don’t know” answers for age, sex, board certification, duration in private practice, number of treated MS patients, and the assumed relevance of the topic pregnancy in MS. Moreover, we evaluated the degree of counseling referring to time and content of counseling.

**Figure 1:**
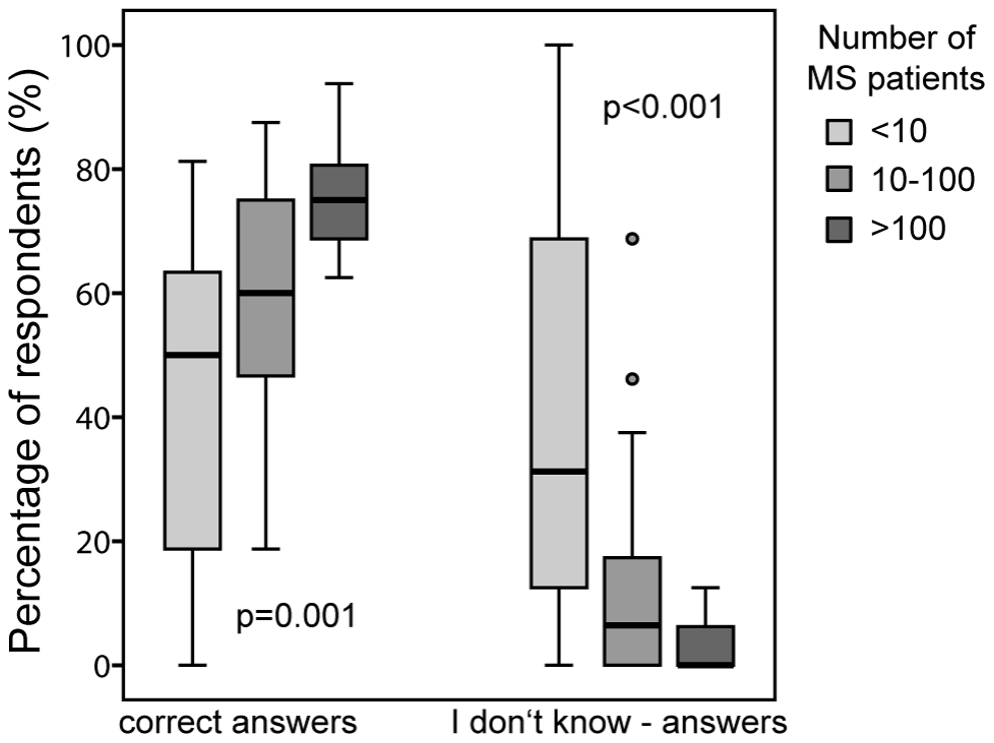
Percentage of correct and I don’t know-answers in relation to the number of treated MS patients per quarter: Kruskal-Wallis-test.

1. *Influence of gender, age and board certification on survey results.* Related to gender, age and the kind of board certification there were no differences in correct and “I don’t know” answers to all questions and to the questions of the various groups.
2. *Influence of period of board certification and duration in private practice on survey results.* If we compare neurologists who passed their exam within the last 15 years to those who are specialized in neurology more than 15 years we found no significant differences in correct and “I don’t know” answers to all questions and to the questions of the various categories. Moreover, there were no differences between physicians in private practice over 10 years compared to neurologists less than 10 years in private practice.
3. *Association between number of treated MS patients and survey results.* The percentage of correct answers to all 16 questions (p = 0.001) and to the questions of category 1 (p<0.001) was significantly associated with the number of treated MS patients. Moreover, the rate of questions answered with “I don’t know” decreases with rising numbers of attended patients for all questions (p = 0.001) and for the questions of the various categories (p<0.001; p = 0.001 and p = 0.01, respectively; Figure 1).
4. *Association between degree of counseling of female patients and survey results.* Significant correlations were found between the degree of counseling and the number of correct answers to all 16 questions (p<0.001) and to questions of the three categories (p<0.001, p = 0.001 and p = 0.004, respectively). Participants that rarely advise patients about pregnancy topics significantly more often answered “I don’t know” to all questions and to questions of the single categories (p<0.001).
5. *Association between degree of counseling of male patients and survey results.* The percentage of correct answers to all 16 questions (p<0.001) and to the questions of the three categories (p<0.001, p = 0.003 and p = 0.03) significantly correlated with the degree of counseling. With rising degree of counseling the rate of questions answered with “I don’t know” decreased for all questions (p = 0.001) and for the questions of the three categories (p = 0.001, p = 0.001 and p<0.001, respectively).
6. *Influence of assumed relevance of pregnancy in MS on survey results.* Physicians that consider pregnancy in MS to be a relevant topic in their practice more frequently answered correctly to all questions (p = 0.002) and to questions of category 1 (p = 0.002) and 2 (p = 0.006). Low assumed relevance is associated with more “I don’t know” answers related to all of the 16 questions (p = 0.001) and to questions of category 1 (p = 0.001), category 2 (p = 0.004) and category 3 (p = 0.014).

**Table 3:** Statistical analysis.

		Correct answers (%)				I Don’t know (%)			
		All questions	category			All questions	category		
			1	2	3		1	2	3
Sex	male	55	54	58	51	21	21	23	22
	female	58	47	69	63	21	28	20	9
	*p-value*	*0.87*	*0.27*	*0.12*	*0.17*	*0.26*	*0.1*	*0.62*	*0.23*
Board certification	neurology	61	55	65	64	19	22	19	10
	neurology & psychiatry	53	48	60	49	23	25	23	22
	*p-value*	*0.27*	*0.31*	*0.67*	*0.83*	*0.86*	*0.74*	*0.97*	*0.08*
Period of board certification	*<15 years*	*56*	*47*	*63*	*62*	*22*	*25*	*23*	*13*
	≥*15 years*	*58*	*55*	*64*	*53*	*20*	*23*	*20*	*17*
	*p-value*	*0.47*	*0.43*	*0.69*	*0.26*	*0.35*	*0.5*	*0.42*	*0.45*
Period in private practice	<10 years	57	51	63	59	23	26	23	17
	≥10 years	56	51	62	52	20	22	20	16
	*p-value*	*0.9*	*0.9*	*0.97*	*0.4*	*0.15*	*0.22*	*0.19*	*0.63*
Treated MS patients per quarter*	<10	42	33	51	47	41	45	40	33
	10–100	59	52	66	60	14	16	14	9
	>100	76	80	76	65	3	3	5	0
	*p-value*	***0.001***	***<0.001***	*0.08*	*0.35*	***<0.001***	***<0.001***	***0.001***	***0.01***
Counseling of female patients about pregnancy related topics*	extensively	69	68	75	57	8	10	7	4
	to a certain degree	53	42	59	64	24	27	24	14
	not at all	11	14	13	0	84	79	83	100
	*p-value*	***<0.001***	***<0.001***	***0.001***	***0.004***	***<0.001***	***<0.001***	***<0.001***	***<0.001***
Counseling of male patients about pregnancy related topics *	extensively	71	73	73	63	8	9	8	3
	to a certain degree	57	48	66	60	18	22	18	10
	not at all	18	18	19	17	73	69	72	83
	*p-value*	***<0.001***	***<0.001***	***0.003***	***0.03***	***0.001***	***0.001***	***0.001***	***<0.001***
Relevance of pregnancy aspects in MS	very/moderately relevant	62	57	69	59	15	17	15	10
	little/not relevant	36	28	41	46	46	50	46	38
	*p-value*	***0.002***	***0.002***	***0.006***	*0.26*	***0.001***	***0.001***	***0.004***	***0.014***

*Kruskal-Wallis-test; for further analysis Man-Whitney-U-test was used.

## Discussion

The intention of the study was to investigate neurologists’ expertise in MS and pregnancy. Overall, respondents answered about half of our questions correctly. Only seven out of 56 (13%) participants indicated not to counsel male and/or female MS patients about pregnancy and sending them to specialized outpatient centers, to a gynecologist, general practitioner or other neurologists. The remaining 49 (87%) physicians reported to counsel MS patients at least to a certain extent. Erroneous knowledge or knowledge gaps revealed by our survey are particularly concerning as they might influence the treatment of patients: for example, 27% of the participants would treat a MS relapse with high dose methylprednisolone (MP) throughout pregnancy. Although administration of MP is not strictly contraindicated during pregnancy, one should be aware that steroids are weak teratogens with higher risks for oral clefts (OR 3.35) [22], miscarriage (12 vs. 7%) [23], and preterm birth (27 vs. 11%) [23] after exposition during the first trimester [22]–[24]. Hence, a strict risk-benefit-assessment is required during the first three months of pregnancy. In contrast, 10% are erroneously convinced that MP is contraindicated throughout the entire pregnancy, although during second and third trimester the use of steroids does not seem to have adverse effects on the fetus [24].

Handling with IFN-β and GA in the context of pregnancy is controversially discussed. According to IFN-β manufacturers’ information, starting of IFN-β therapy during pregnancy is contraindicated. Likewise, it is common practice to discontinue IFN-β or GA treatment when a pregnancy is detected. On the other hand, the manufacturers indicate that in highly active MS IFN-β discontinuation during pregnancy should be critically weighted up against the risk of severe relapses and there are several reports showing no detrimental effects when IFN-β and GA were continued during pregnancy [25], [26]. This view, however, conflicts with Lu et al. [27], who in general recommend discontinuation of DMT when a pregnancy is planned. This recommendation is based on one single study where birth weight and length were reduced on average by 114 g and 1.1 cm, respectively [28]. However, unlike mitoxantrone, no precise interval between withdrawal of IFN-β and begin of unprotected sexual intercourse is defined in women planning to conceive. On the contrary, some authors warn of leaving patients untreated while waiting for conception [29].

Prevalence of sexual dysfunction is underestimated by many physicians. 50% of respondents think that only 10 or 40% of MS patients were affected by symptoms like reduced libido, erectile dysfunction or disturbances of ejaculation. However, recent studies showed that up to 70% of MS patients suffer from sexual dysfunctions [30], [31]. Moreover, 20% respectively 8% of respondents erroneously think that a vaginal delivery mode or a peridural anesthesia is responsible for higher relapse rate after delivery. That is a cause of concern as thereby unnecessarily a primary caesarian section may be recommended or patients unnecessarily avoid peridural anesthesia. The questions most often answered with “I don’t know” refer to assisted delivery and artificial fertilization which reflects the necessity of close cooperation with obstetricians. Overall, a fifth (21%) of all questions were answered with “I don’t know”.

Most knowledge gaps concern questions of category 1 dealing with family planning before conception. This is a cause of concern as incorrect advice at this time may have devastating consequences such as abstaining from having children at all, or abortion or congenital malformations due to certain medications. The percentage of correct answers significantly increases when more than 100 MS patients were treated per quarter (≙ 400 MS patients per year).

Until now, the only study which investigated neurologists’ treatment regimes about gender specific topics in MS was performed by Coyle et al [32]. In that study, which was reported in 2004, up to 79% of respondents recommended discontinuing IMT prior to planned conception, whereas in our study only 27% counsel in this way. In our study 50% gave advice for discontinuing IMT only after conception, compared to 20% in the other study. These differences probably reflect the changes in treatment strategies due to the increase of knowledge over the last ten years. Further questions in the study by Coyle et al. [32] refer to contraception, menstrual irregularities, hormone replacement therapy, and breastfeeding. Other relevant topics such as treatment of relapses during pregnancy and lactation, mode of delivery, or risks of assisted reproductive technique were not discussed. Another limitation of that study is that only female neurologists were included. The surveyed cohort was less homogenous than ours, as the interviewed physicians worked as well in private practice as in academic and MS centers in the USA and Canada. In our study only physicians in private practice in Berlin were surveyed.

Our study has a number of limitations as well. As response rate was only 23% a non-response bias cannot be ruled out. However, previous questionnaire studies among physicians showed only few differences between responders and non-responders [33]–[35] suggesting that nonresponse bias may not be as crucial as in surveys of the general population. Since only city-based physicians in private practice were included it is impossible to estimate to what degree our results are representative of other regions or countries. Berlin is Germany’s largest city, and supposedly, the overall offer for education is higher than in rural areas. Therefore it cannot be ruled out that our survey performed in rural areas might have resulted in a lower proportion of correct answers. Although our findings are not applicable to rural areas, it is possible that our results even overestimate physicians’ expertise in general. Moreover, compared to neurologists in private practice, hospital-based physicians and neurologists in other institutions may differ in their expertise about pregnancy aspects in MS. To address these issues, a survey of neurologists outside Berlin as well as of physicians in academic centers and the comparison to neurologists in private practice might be the objective of further studies.

One fifth of the participants in our study do not consider pregnancy to be a relevant topic in their practice. We assume that those physicians who answered the questionnaire are in general more interested in the topic than non-responders. Thus, it is possible that expertise in general may be lower than determined in our study. Likewise, we cannot rule out that respondents looked up the relevant information which could also result in overestimation of expertise.

To sum up, our results provide some insights into the state of knowledge among neurologists in private practice in Berlin, Germany. MS patients cannot take for granted to receive correct advice concerning pregnancy related topics. Level of expertise rises with the number of attended MS patients and assumed relevance of the topic. Therefore MS patients should seek advice from physicians attending a large number of MS patients. Many uncertainties regarding assisted delivery and artificial fertilization reveals the importance of close cooperation with attending obstetricians.

## Supporting Information

Questionnaire S1 Multiple sclerosis (MS), family planning and pregnancy.
(DOC)

## References

[1] Pugliatti M, Rosati G, Carton H, Riise T, Drulovic J, et al (2006) The epidemiology of multiple sclerosis in Europe. Eur J Neurol Off J Eur Fed Neurol Soc 13: 700–722 10.1111/j.1468-1331.2006.01342.x 16834700

[2] Koutsouraki E, Costa V, Baloyannis S (2010) Epidemiology of multiple sclerosis in Europe: a review. Int Rev Psychiatry Abingdon Engl 22: 2–13 10.3109/09540261003589216 20233110

[3] Kotzamani D, Panou T, Mastorodemos V, Tzagournissakis M, Nikolakaki H, et al (2012) Rising incidence of multiple sclerosis in females associated with urbanization. Neurology 78: 1728–1735 10.1212/WNL.0b013e31825830a9 22592376

[4] Orton S-M, Herrera BM, Yee IM, Valdar W, Ramagopalan SV, et al (2006) Sex ratio of multiple sclerosis in Canada: a longitudinal study. Lancet Neurol 5: 932–936 10.1016/S1474-4422(06)70581-6 17052660

[5] Trojano M, Lucchese G, Graziano G, Taylor BV, Simpson S Jr, et al (2012) Geographical variations in sex ratio trends over time in multiple sclerosis. PloS One 7: e48078 10.1371/journal.pone.0048078 23133550 PMC3485003

[6] Schapira K, Poskanzer DC, Newell DJ, Miller H (1966) Marriage, pregnancy and multiple sclerosis. Brain J Neurol 89: 419–428. 10.1093/brain/89.3.419 5921125

[7] Chen YH, Lin HL, Lin HC (2009) Does multiple sclerosis increase risk of adverse pregnancy outcomes? A population-based study. Mult Scler Houndmills Basingstoke Engl 15: 606–612 10.1177/1352458508101937 19318510

[8] Dahl J, Myhr K-M, Daltveit AK, Hoff JM, Gilhus NE (2005) Pregnancy, delivery, and birth outcome in women with multiple sclerosis. Neurology 65: 1961–1963 10.1212/01.wnl.0000188898.02018.95 16380620

[9] Finkelsztejn A, Brooks JBB, Paschoal FM Jr, Fragoso YD (2011) What can we really tell women with multiple sclerosis regarding pregnancy? A systematic review and meta-analysis of the literature. BJOG Int J Obstet Gynaecol 118: 790–797 10.1111/j.1471-0528.2011.02931.x 21401856

[10] Van der Kop ML, Pearce MS, Dahlgren L, Synnes A, Sadovnick D, et al (2011) Neonatal and delivery outcomes in women with multiple sclerosis. Ann Neurol 70: 41–50 10.1002/ana.22483 21710652 PMC3625744

[11] Jalkanen A, Alanen A, Airas L (2010) Finnish Multiple Sclerosis and Pregnancy Study Group (2010) Pregnancy outcome in women with multiple sclerosis: results from a prospective nationwide study in Finland. Mult Scler Houndmills Basingstoke Engl 16: 950–955 10.1177/1352458510372629 20542921

[12] Mueller BA, Zhang J, Critchlow CW (2002) Birth outcomes and need for hospitalization after delivery among women with multiple sclerosis. Am J Obstet Gynecol 186: 446–452. 11904605 10.1067/mob.2002.120502

[13] Confavreux C, Hutchinson M, Hours MM, Cortinovis-Tourniaire P, Moreau T (1998) Rate of pregnancy-related relapse in multiple sclerosis. Pregnancy in Multiple Sclerosis Group. N Engl J Med 339: 285–291 10.1056/NEJM199807303390501 9682040

[14] Calabrò RS, De Luca R, Conti-Nibali V, Reitano S, Leo A, et al (2013) Sexual Dysfunction in Male Patients with Multiple Sclerosis: A Need for Counseling! Int J Neurosci. 10.3109/00207454.2013.865183 24219384

[15] Borisow N, Döring A, Pfueller CF, Paul F, Dörr J, et al (2012) Expert recommendations to personalization of medical approaches in treatment of multiple sclerosis: an overview of family planning and pregnancy. EPMA J 3: 9 10.1186/1878-5085-3-9 22738272 PMC3464716

[16] Sandberg-Wollheim M, Alteri E, Moraga MS, Kornmann G (2011) Pregnancy outcomes in multiple sclerosis following subcutaneous interferon beta-1a therapy. Mult Scler Houndmills Basingstoke Engl 17: 423–430 10.1177/1352458510394610 21220368

[17] Weber-Schoendorfer C, Schaefer C (2009) Multiple sclerosis, immunomodulators, and pregnancy outcome: a prospective observational study. Mult Scler Houndmills Basingstoke Engl 15: 1037–1042 10.1177/1352458509106543 19692433

[18] Hoevenaren IA, de Vries LC, Rijnders RJP, Lotgering FK (2011) Delivery of healthy babies after natalizumab use for multiple sclerosis: a report of two cases. Acta Neurol Scand 123: 430–433 10.1111/j.1600-0404.2010.01426.x 21492099

[19] Hellwig K, Haghikia A, Gold R (2011) Pregnancy and natalizumab: results of an observational study in 35 accidental pregnancies during natalizumab treatment. Mult Scler Houndmills Basingstoke Engl 17: 958–963 10.1177/1352458511401944 21613333

[20] Hellwig K, Haghikia A, Agne H, Beste C, Gold R (2009) Protective effect of breastfeeding in postpartum relapse rate of mothers with multiple sclerosis. Arch Neurol 66: 1580–1581; 10.1001/archneurol.2009.281 20008670

[21] Portaccio E, Ghezzi A, Hakiki B, Martinelli V, Moiola L, et al (2011) Breastfeeding is not related to postpartum relapses in multiple sclerosis. Neurology 77: 145–150 10.1212/WNL.0b013e318224afc9 21734184

[22] Park-Wyllie L, Mazzotta P, Pastuszak A, Moretti ME, Beique L, et al (2000) Birth defects after maternal exposure to corticosteroids: prospective cohort study and meta-analysis of epidemiological studies. Teratology 62: 385–392 doi:;10.1002/1096-9926(200012)62:6<385::AID-TERA5>3.0.CO;2-Z 11091360 10.1002/1096-9926(200012)62:6<385::AID-TERA5>3.0.CO;2-Z

[23] Gur C, Diav-Citrin O, Shechtman S, Arnon J, Ornoy A (2004) Pregnancy outcome after first trimester exposure to corticosteroids: a prospective controlled study. Reprod Toxicol Elmsford N 18: 93–101 10.1016/j.reprotox.2003.10.007 15013068

[24] Ferrero S, Pretta S, Ragni N (2004) Multiple sclerosis: management issues during pregnancy. Eur J Obstet Gynecol Reprod Biol 115: 3–9 10.1016/j.ejogrb.2003.10.020 15223156

[25] Hellwig K, Gold R (2011) Glatiramer acetate and interferon-beta throughout gestation and postpartum in women with multiple sclerosis. J Neurol 258: 502–503 10.1007/s00415-010-5758-2 20878174

[26] Dung Dung AA, Panda AK (2014) Interferon β-1a therapy for multiple sclerosis during pregnancy: an unresolved issue. BMJ Case Rep 2014 10.1136/bcr-2013-201273 PMC3987290 24711465

[27] Lu E, Wang BW, Guimond C, Synnes A, Sadovnick D, et al (2012) Disease-modifying drugs for multiple sclerosis in pregnancy: a systematic review. Neurology 79: 1130–1135 10.1212/WNL.0b013e3182698c64 22933738 PMC3525300

[28] Amato MP, Portaccio E, Ghezzi A, Hakiki B, Zipoli V, et al (2010) Pregnancy and fetal outcomes after interferon-β exposure in multiple sclerosis. Neurology 75: 1794–1802 10.1212/WNL.0b013e3181fd62bb 21079181

[29] Charles JA, Tremlett H, Lu E, Guimond C, Sadovnick AD (2013) Disease-modifying drugs for multiple sclerosis in pregnancy: a systematic review. Neurology 80: 1068–1069 10.1212/01.wnl.0000428359.07207.f6 23479468

[30] Zorzon M, Zivadinov R, Bosco A, Bragadin LM, Moretti R, et al (1999) Sexual dysfunction in multiple sclerosis: a case-control study. I. Frequency and comparison of groups. Mult Scler Houndmills Basingstoke Engl 5: 418–427. 10.1177/135245859900500i609 10618699

[31] Celik DB, Poyraz EÇ, Bingöl A, Idiman E, Ozakbaş S, et al (2013) Sexual dysfunction ın multiple sclerosis: gender differences. J Neurol Sci 324: 17–20 10.1016/j.jns.2012.08.019 23079605

[32] Coyle PK, Christie S, Fodor P, Fuchs K, Giesser B, et al (2004) Multiple sclerosis gender issues: clinical practices of women neurologists. Mult Scler Houndmills Basingstoke Engl 10: 582–588. 10.1191/1352458504ms1083oa 15471377

[33] McFarlane E, Olmsted MG, Murphy J, Hill CA (2007) Nonresponse bias in a mail survey of physicians. Eval Health Prof 30: 170–185 10.1177/0163278707300632 17476029

[34] Hovland EJ, Romberg E, Moreland EF (1980) Nonresponse bias to mail survey questionnaires within a professional population. J Dent Educ 44: 270–274. 6928881

[35] Kellerman SE, Herold J (2001) Physician response to surveys. A review of the literature. Am J Prev Med 20: 61–67. 10.1016/s0749-3797(00)00258-0 11137777

