# Supplementary material for: Pregnancy in Multiple Sclerosis: A Questionnaire Study
Source: PLoS One. 2014 Jun 5;9(6):e99106. doi: 10.1371/journal.pone.0099106 (PMC4047092; doi:10.1371/journal.pone.0099106)
Supplement: Questionnaire S1 — Multiple sclerosis (MS), family planning and pregnancy. (DOC) [file pone.0099106.s001.doc]

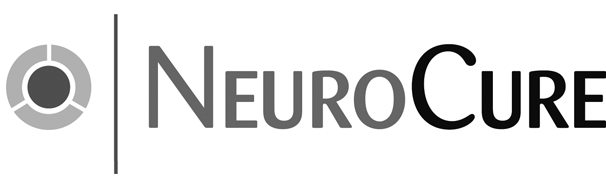

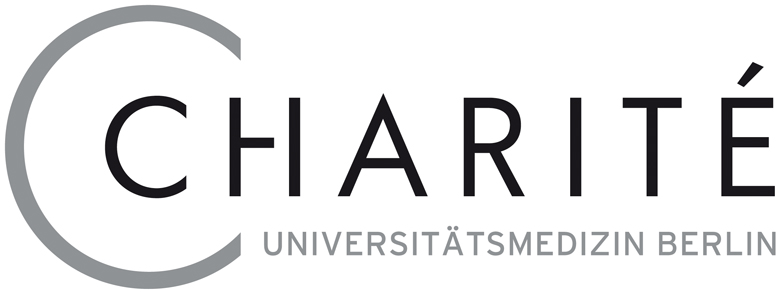


**Questionnaire**

**“Multiple sclerosis (MS), family planning and pregnancy“**

**Instructions for completing the questionnaire:** Please answer every question. Please use a black or blue pen, mark the correct answer clearly with a tick and tick one box only. In the case that you cannot answer a specific question, please do not guess but tick “I don´t know”. Thank you very much.

**Part 1 Demographic data**

| ***Please tick one box only!*** | | | | |
| --- | --- | --- | --- | --- |
| Age | ____________ (years) | | | |
| Sex | □ male | | □ female | |
| Qualification | □ neurology | | □ neurology & psychiatry | |
| Date of board certicication | _____________(year) | | | |
| Private practice since | _____________(year) | | | |
| On average, how many MS patients do you treat per quarter (3 months)? | □< 10 | □10-100 | | □>100 |
| Do you counsel female MS patients about family planning and pregnancy? | □yes, extensively  □yes, to a certain degree  □no, I send the patient to _______________  (specialisation) | | | |
| Do you counsel male MS patients about family planning and pregnancy? | □yes, extensively  □yes, to a certain degree  □no, I send the patient to _______________  (specialisation) | | | |
| How relevant are pregnancy related topics in MS in your practice? | □very relevant  □moderately relevant  □little relevant  □not relevant | | | |

| **Part 2 Questionnare** |
| --- |
|  |
| 1. Compared to general population, how much higher is MS risk in children, when one parent is suffering from MS?  - Not increased - 6-12 times higher - 20-30 times higher - I don´t know. |
| 1. What proportion of MS patients approximately suffer from sexual dysfunctions (e.g. reduced libido, erectile or ejaculation dysfunctions, difficulties in reaching orgasm)?  - 10% - 40% - 70% - I don´t know. |
| 1. Which recommendation about basic immunomodulatory therapy would you give to female MS patients with desire for children?  - Interferon (IFN) and glatiramer acetate (GA) should be discontinued three month before a planned conception. - Application of IFN and GA during conception and the first weeks of pregnancy should be discontinued when pregnancy is detected. - Application of IFN and GA can be continued without any concern for the complete duration of pregnancy. - I don´t know. |
| 1. Which recommendation do you offer male MS patients with desire for children?  - Interferon (IFN) and glatiramer acetate (GA) should be discontinued three month before a planned conception. - IFN and GA should be discontinued at the beginning of unprotected sexual intercourse. - Application of IFN and GA can be continued while having unprotected sexual intercourse. - I don´t know. |
| 1. How do you council child bearing MS patients with escalation therapy?  - Before a planned pregnancy natalizumab has to be discontinued as it is proven to significantly increase risk of congenital malformations. - As patients with fingolimod usually have a high disease activity, female patients should continue intake of fingolimod medication during the entire pregnancy. - Due to its potential teratogenicity, mitoxantrone should be withdrawn six months before a planned pregnancy in male as well as in female patients. - I don´t know. |
| 1. How does pregnancy influences MS relapse rate?  - Relapse rate decreases during the course of pregnancy. - Relapse rate remains unchanged. - Relapse rate increases during the course of pregnancy. - I don´t know. |

| **Part 2 Questionnaire** |
| --- |
|  |
| 1. Which information do you offer MS patients about possible risks and complications during pregnancy?  - Complications as (pre-) eclampsia or gestational diabetes occur less often compared to women without MS. - Rate of pregnancy complications is comparable with that of healthy pregnant women. - Compared to healthy pregnant women, MS patients have increased complications during pregnancy. - I don´t know. |
| 1. Would you treat a relapse in a pregnant MS patient with steroids (1 g methylprednisolone i.v. for 3-5 days)?  - Yes, even during pregnancy there is no limitation to treat relapses with methylprednisolone. - High dose steroids are only safe during second and third trimester. - No, administration of methylprednisolone is contraindicated during the entire pregnancy. - I don´t know. |
| 1. Which statement about assisted delivery in MS patients is correct?  - Compared to healthy women use of forceps or vacuum extraction is needed more often in MS patients. - To avoid complications a caesarean section should be generally recommended in MS patients. - Compared to caesarean section vaginal delivery leads to increased postpartum relapse rate due to increased release of adrenalin and noradrenalin during delivery. - I don´t know. |
| 1. What information do you offer MS patients regarding use of peridural anesthesia (PDA)?  - PDA should be avoided in MS patients as it leads to higher rates of delivery complications. - Patients should be informed that PDA can increase disease activity within the first month after child birth. - PDA has no influence on postpartum relapse rate and can be used in MS patients, too. - I don´t know. |
| 1. Which statement about outcome of pregnancies in MS patients (without disease modifying therapy) is correct?  - There is no difference in risk of congenital malformations compared to pregnancies of healthy women. - Newborns of MS patients have lower birth weight compared to newborns of healthy women. - There is a higher risk of spontaneous abortion and preterm birth in MS patients. - I don´t know. |

| **Part 2 Questionnaire** |
| --- |
|  |
| 1. Which information can you offer about post partum relapse rate?  - Relapse rate is lower than before pregnancy. - Relapse rate is unchanged compared to the period before pregnancy. - Relapse rate is higher than before pregnancy. - I don´t know. |
| 1. With respect to MS disease, would you recommend breastfeeding?  - Yes, because it is proven that breastfeeding reduces disease activity and postpartum relapse rate. - No, because breastfeeding increases postpartum relapse rate. - According to the current state of data effect of breastfeeding on relapse rate is not finally clarified. That is why I could not give any specific recommendation. - I don´t know. |
| 1. Which recommendation for MS therapy during breastfeeding would you give?  - To treat an acute MS relapse with methylprednisolone, it is necessary to immediately stop breastfeeding. - After childbirth early resumption of immunomodulatory therapy (IMT) is possible and necessary, even if the women are breastfeeding. - During period of breastfeeding you have to weigh up early resumption of IMT on the one hand and potentially beneficial effects of breastfeeding on the other hand. - I don´t know. |
| 1. To what degree do pregnancies or deliveries influence the long term course of MS?  - Previous studies could not clearly show neither protective nor negative effects on long term MS course. - According to long term course of MS, pregnancies increase disease activity resulting in a higher degree of disability. - Pregnancies have a positive effect on long term MS disease course and lead to a decrease of disease progression. - I don´t know. |
| 1. Which statement about assisted reproduction technique is correct?  - Use of assisted reproduction technique is contraindicated in MS. - Patients should be informed about increased risk of relapses due to hormonal treatment in the context of artificial fertilization. - Administration of IFN oder GA should be discontinued at the latest when starting assisted reproduction technique. - I don´t know. |

**We thank you for your cooperation!**
